# Artificial Intelligence Anxiety Levels Among Healthcare Professionals: A Systematic Review and Meta‐Analysis

**DOI:** 10.1155/jonm/5410088

**Published:** 2026-06-02

**Authors:** Hilal Kahraman, Hatice Yüceler Kaçmaz, Seda Akutay

**Affiliations:** ^1^ Faculty of Health Sciences, Department of Surgical Nursing, University of Erciyes, Kayseri, Türkiye, erciyes.edu.tr

**Keywords:** anxiety, artificial intelligence, artificial intelligence anxiety, healthcare professionals

## Abstract

**Background:**

Artificial intelligence (AI) is increasingly being integrated into various aspects of healthcare delivery, from diagnostics to patient management. While these advancements offer significant benefits, they also raise anxiety among healthcare professionals regarding job security, ethical implications, and changes in clinical decision‐making. Despite the growing importance of this issue, there is limited consensus among healthcare professionals regarding the overall level of anxiety about AI.

**Methods:**

This systematic review and meta‐analysis was conducted to assess healthcare professionals’ anxiety about AI. This study was conducted in accordance with PRISMA guidelines and registered in the PROSPERO database (CRD420251017693). A comprehensive literature search was conducted in the Web of Science, PubMed, Scopus, EBSCO, ScienceDirect, and Wiley Online Library databases up to April 2025. Studies focusing on healthcare professionals’ levels of anxiety about AI were included. Risk of bias was assessed using the JBI critical appraisal tool. In the analyses, effect sizes were calculated using the random effects model, while heterogeneity, sensitivity, and publication bias were assessed via Cochrane’s Q, *I* ^2^, *τ* ^2^, leave‐one‐out analysis, funnel plots, and Egger’s and Begg’s tests.

**Results:**

Nine studies involving 1877 healthcare professionals were included in the systematic review, and five studies (*n* = 926) that met the criteria were included in the meta‐analysis. The meta‐analysis revealed that the mean score on the AI anxiety scale was 59.26 (95% CI = 40.54–77.97), indicating that healthcare professionals generally experience a moderate level of AI‐related anxiety. A statistically significant heterogeneity was observed among the included studies (*I* ^2^ = 99.1%, *τ* ^2^ = 224.20, *p* < 0.0001).

**Conclusion:**

While it can be suggested that healthcare professionals experience moderate to high levels of anxiety towards AI technologies, the high heterogeneity observed across the included studies makes it difficult to draw a generalized conclusion. The study highlights the necessity of proactive measures, including education and organizational support, to build healthcare professionals’ confidence and readiness for AI adoption.

## 1. Introduction

Artificial intelligence (AI) refers to a broad domain focused on enabling computer systems to simulate human capabilities such as reasoning, communication, and decision‐making [1–3]. The foundations of AI date back to the 1950s; however, since 2011, AI has advanced rapidly due to improvements in computing capabilities and the emergence of large datasets that facilitate the training of AI systems. This rapid advancement of AI has also been reflected in healthcare, where it is being utilized in various domains such as diagnosis, medical imaging, decision support, disease risk prediction, surgical procedures, patient care, medication tracking, health education, and appointment scheduling systems [4–7]. The increasing use of AI in healthcare contributes to overall service efficiency by automating routine workflows, reducing the workload of healthcare professionals, and facilitating time management [8, 9].

All these advancements have accelerated and expanded the use of AI within healthcare systems. However, the rapid integration of AI‐based applications into healthcare services has raised important anxiety that healthcare professionals may face various challenges adapting to these emerging technologies. In this review, the term “healthcare professionals” refers to licensed health personnel involved in patient care within healthcare systems, including physicians, academicians, nurses, midwives, dentists, pharmacists, physiotherapists, dietitians, and psychologists. Individuals who had not yet graduated from these professions (e.g., undergraduate students) were excluded from this definition. Özçevik Subaşı et al. [10] noted that healthcare professionals’ attitudes toward AI have not consistently developed positively [10]. The integration of AI into decision‐making processes may lead to anxiety among some healthcare professionals regarding the potential devaluation of their professional roles, reduced job security, or the marginalization of their clinical decision‐making skills [5, 11, 12]. Moreover, changes in the roles and responsibilities of healthcare professionals, lack of technological infrastructure, limited training opportunities, and ethical anxiety related to AI are among the factors that exacerbate these anxieties. This situation is considered not only individual‐level resistance but also a structural barrier that can directly impact the digital transformation process of healthcare services [3, 13]. Therefore, studies aimed at understanding healthcare professionals’ anxieties toward AI are critically important for the successful and sustainable integration of the technology.

AI refers to a broad domain focused on enabling computer systems to simulate human capabilities such as reasoning, communication, and decision‐making [1–3]. The foundations of AI date back to the 1950s; however, since 2011, AI has advanced rapidly due to improvements in computing capabilities and the emergence of large datasets that facilitate the training of AI systems. This rapid advancement of AI has also been reflected in healthcare, where it is being utilized in various domains such as diagnosis, medical imaging, decision support, disease risk prediction, surgical procedures, patient care, medication tracking, health education, and appointment scheduling systems [4–7]. The increasing use of AI in healthcare contributes to overall service efficiency by automating routine workflows, reducing the workload of healthcare professionals, and facilitating time management [8, 9].

All these advancements have accelerated and expanded the use of AI within healthcare systems. However, the rapid integration of AI‐based applications into healthcare services has raised important anxiety that healthcare professionals may face various challenges adapting to these emerging technologies. Özçevik Subaşı et al. [10] noted that healthcare professionals’ attitudes toward AI have not consistently developed positively [10]. The integration of AI into decision‐making processes may lead to anxiety among some healthcare professionals regarding the potential devaluation of their professional roles, reduced job security, or the marginalization of their clinical decision‐making skills [5, 11, 12]. Moreover, changes in the roles and responsibilities of healthcare professionals, lack of technological infrastructure, limited training opportunities, and ethical anxiety related to AI are among the factors that exacerbate these anxieties. This situation is considered not only as individual‐level resistance but also as a structural barrier that can directly impact the digital transformation process of healthcare services [3, 13]. Therefore, studies aimed at understanding healthcare professionals’ anxieties toward AI are critically important for the successful and sustainable integration of the technology.

Despite the increasing number of studies addressing AI in healthcare, the literature examining AI‐related anxiety among healthcare professionals remains limited and methodologically heterogeneous. Many of the existing studies have primarily focused on attitudes toward AI, technology acceptance, or digital readiness rather than directly examining anxiety as a distinct psychological construct [14–16]. In addition, most studies have employed cross‐sectional designs conducted within single institutions or specific professional groups, which restricts the generalizability of their findings [10, 17–19]. Furthermore, considerable variability exists in the measurement tools used to assess AI‐related anxiety, ranging from general technology‐related anxiety scales to newly developed instruments specific to AI, making comparisons across studies difficult [10, 19–22]. These methodological and conceptual differences result in fragmented evidence and limit the ability to obtain a comprehensive understanding of the overall level of AI‐related anxiety among healthcare professionals across different healthcare contexts.

A systematic review of the existing literature is essential to understand and manage healthcare professionals’ anxieties related to AI. The literature includes studies investigating the anxiety levels of healthcare professionals and the associated factors [10, 17, 22]. Although individual studies conducted in the field shed light on the topic, significant differences can be observed among their findings due to variations in professional groups, geographical regions, and measurement instruments used. This situation reduces the generalizability of the presented information and complicates reaching common conclusions. Therefore, systematically compiling and analyzing studies on the subject from a comprehensive perspective will contribute both to the advancement of the research field and to providing guiding data for key stakeholders involved in healthcare planning. The primary aim of this study is to provide a comprehensive perspective on the field by systematically examining healthcare professionals’ anxiety levels related to AI based on the existing literature.

## 2. Materials and Methods

### 2.1. Study Design

This review was planned and conducted by the Preferred Reporting Items for Systematic Reviews and Meta‐Analyses (PRISMA) guidelines, which aim to enhance methodological transparency in systematic review and meta‐analysis studies. The review protocol was registered in the PROSPERO database, an international registry for systematic review protocols (CRD420251017693), and no amendments were made to the protocol throughout the duration of the study.

### 2.2. Research Question

What is the level of anxiety experienced by healthcare professionals in relation to AI?

### 2.3. Search Strategy and Study Selection

The basic search strategy was structured using the Population, Intervention, Comparison, Outcomes (PICO) approach, which aids in systematically and precisely defining the research question. The study population (*p*) consists of healthcare professionals. Studies aimed at determining AI anxiety levels were evaluated as the intervention (I). No comparison (C) was included in the review, while the outcome (O) was the anxiety levels of healthcare professionals toward AI (Table 1). This framework enabled a systematic literature search and clearly delineated the scope of the study.

**TABLE 1:** PICO framework of the meta‐analysis.

*P* (population)	Health professionals
I (Intervention)	Studies determining artificial intelligence anxiety level
C (Comparison)	Artificial Intelligence
O (Outcome)	Anxiety

Within the scope of this systematic review, a literature search was conducted covering the period from the inception of the databases up to April 2025. The literature search utilized the following online databases: Web of Science, PubMed, Scopus, EBSCO, ScienceDirect, and Wiley Online Library. The search strategy was developed by combining free‐text terms and their synonyms, structured using the Boolean operators “AND” and “OR.” The main search terms employed in the literature review were as follows: “health professionals” OR “health care professional” OR “health personnel” OR “healthcare provider” OR “healthcare worker” OR “nurse” OR “midwife” OR “physician” OR “doctor” OR “physiotherapist” OR “dietician” OR “pharmacist” OR “psychologist” AND “Artificial Intelligence” OR “AI” AND “anxiety.”

### 2.4. Inclusion and Exclusion Criteria

The inclusion criteria were as follows: (1) Type of study: peer‐reviewed original research, including quantitative studies (such as descriptive, cross‐sectional, and randomized controlled trials) and mixed‐method studies that reported quantitative data related to AI anxiety; (2) Participants: health professionals; (3) Intervention: studies assessing AI anxiety among health professionals using the AI anxiety scale (AIAS); (4) Language: studies published in English; and (5) Accessibility: studies with full‐text availability. Interventional studies were considered eligible if they reported measurable outcomes related to AI anxiety among health professionals. For mixed‐methods studies, only the quantitative components relevant to AI anxiety outcomes were extracted and included in the analysis. The exclusion criteria were (1) studies assessing anxiety outcomes in populations other than health professionals and (2) nonoriginal research such as systematic reviews, meta‐analyses, narrative reviews, scoping reviews, editorials, commentaries, and conference proceedings.

### 2.5. AIAS

The AIAS was developed by Wang and Wang in 2019 to measure anxiety related to AI [23]. The scale has been used in Indonesian, Turkish, and German languages [22, 24–26]. In the studies included in the meta‐analysis, both the original 21‐item scale with completed validity and reliability studies [26] and the 16‐item version developed following validity and reliability assessments [24] were considered. The scale consists of four subdimensions: learning, job loss, sociotechnical blindness, and AI configuration. The learning subdimension reflects individuals’ anxiety about using AI in their professional development. The job loss subdimension represents the anxiety that the advancement and integration of AI into the profession will reduce or eliminate the need for their roles, leading to fears of unemployment. The sociotechnical blindness subdimension reflects beliefs regarding individuals’ lack of knowledge about AI applications. The AI configuration subdimension captures anxiety stemming from perceiving AI applications as frightening. It is noted that AI anxiety may vary among individuals, potentially producing positive effects in some while adversely affecting behaviors in others. An increase in the scale score indicates a higher level of AI‐related anxiety [23, 26]. The use of the AIAS was considered within the inclusion criteria in order to enhance methodological consistency and ensure the standardization and comparability of the results across studies.

### 2.6. Data Extraction

Following the database search, the data were uploaded to the Covidence software. After removing duplicate publications through Covidence, two researchers (HK, HYK) independently screened the titles and abstracts of all articles submitted to the software for eligibility criteria. Studies lacking sufficient information in the abstract regarding eligibility criteria were subjected to full‐text review for more detailed assessment. Full texts of studies potentially eligible for inclusion in the systematic review were obtained and evaluated in terms of relevance according to the predefined eligibility criteria. After the review process, data extraction was independently performed by two researchers (HK, HYK) for the studies to be included. In case of discrepancies, disagreements were resolved by consensus with the input of a third author (SA).

### 2.7. Quality and Bias Assessment

Two researchers (HK, HYK) independently conducted quality assessments of the included studies. This assessment was conducted to evaluate the methodological quality of the studies and to determine the extent to which potential biases in their design, conduct, and analysis were addressed. The Joanna Briggs Institute (JBI) tool was used to assess the quality of the cross‐sectional studies included in the review, employing eight questions for evaluation [27]. Although one of the included studies was a mixed‐methods study [22], since the qualitative and quantitative data were presented as two separate datasets, the quantitative data were also assessed using the eight‐item JBI tool designed for cross‐sectional studies [28]. One of the included studies was quasi‐experimental, and for this study, the nine‐item JBI tool developed specifically for quasi‐experimental studies was used [29]. The ratio of items answered ‘yes’ to the total number of items was calculated for each study, and the resulting percentage was determined. Based on this assessment, studies scoring between 0% and 49% were classified as low quality, 50% and 69% as moderate quality, and those scoring ≥ 70% as high quality [30] (Table 2).

**TABLE 2:** JBI tools for the quality assessment.

JBI items (cross‐sectional)/studies	[20]	[21]	[17]	[10]	[33]	[19]	[22]	[34]	JBI items (quasi‐experimental)/study	[32]
Were the criteria for inclusion in the sample clearly defined?	N	N	Y	Y	N	Y	Y	N	Is it clear in the study what is the “cause” and what is the “effect”?	Y
Were the study subjects and the setting described in detail?	Y	Y	Y	Y	Y	Y	Y	Y	Was there a control group?	N
Was the exposure measured in a valid and reliable way?	Y	Y	Y	Y	Y	Y	Y	Y	Were participants included in any comparisons similar?	Y
Were objective, standard criteria used for measurement of the condition?	Y	Y	Y	Y	Y	Y	Y	Y	Were the participants included in any comparisons receiving similar treatment/care, other than the exposure or intervention of interest?	N
Was confounding factors identified?	N	N	N	N	N	N	N	N	Were there multiple measurements of the outcome, both pre and post the intervention/exposure?	Y
Were strategies to ideal with confounding factors stated?	N	N	N	N	N	N	N	N	Were the outcomes of participants included in any comparisons measured in the same way?	Y
Were the outcomes measured in a valid and reliable way?	Y	Y	Y	Y	Y	Y	Y	Y	Were outcomes measured in a reliable way?	Y
Was appropriate statistical analysis used?	Y	Y	Y	Y	Y	Y	U	Y	Was follow‐up complete and if not, were differences between groups in terms of their follow‐up adequately described and analyzed?	Y
									Was appropriate statistical analysis used?	Y
**Total**	5/8	5/8	6/8	6/8	5/8	6/8	5/8	5/8	**Total**	7/9
**Quality score**	**62.5%**	**62.5%**	*75%*	*75%*	**62.5%**	*75%*	62.5%	62.5%	**Quality score**	*77.7%*

*Note:* Y: yes, N: no, U: unclear. Italics: high quality, Bold: moderate quality.

### 2.8. Statistical Analysis

The systematic review component of this study employed a narrative synthesis approach, in which the findings of the included studies were summarized and interpreted descriptively without statistical pooling. The meta‐analyses in this study were conducted using *R* Studio (version 4.5.0) with the meta and dplyr packages. Separate analyses were performed for the total scores and subdimensions of the AIAS. For each study, analyses were performed using the meta mean function based on sample size, mean, and standard deviation values; raw mean values (MRAW) were calculated as the effect size. Effect sizes were interpreted based on the scoring range of the AIAS, where higher mean scores indicate greater levels of AI‐related anxiety. Considering the clinical heterogeneity among the data, a random‐effects model was applied in all analyses. Heterogeneity was assessed using Cochrane’s Q test (Q), the I‐squared statistic (*I* ^2^), and Tau‐squared (*τ* ^2^). Heterogeneity was interpreted according to conventional thresholds, whereby *I* ^2^ values of 25%, 50%, and 75% were considered to indicate low, moderate, and high heterogeneity, respectively [31]. Forest plots display the individual mean values and 95% confidence intervals for each study. Sensitivity analysis was conducted using the leave‐one‐out method, where one study is excluded at a time to assess the robustness and stability of the pooled estimates. Finally, publication bias was assessed to evaluate the study’s validity using funnel plots and Egger’s and Begg’s tests, with a *p* value of less than 0.05 considered indicative of statistically significant publication bias.

## 3. Results

### 3.1. Search Results

**FIGURE 1:**
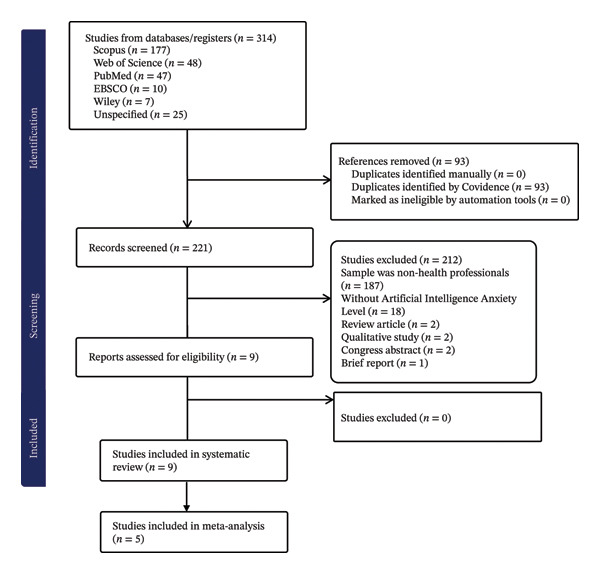
PRISMA 2020 flow diagram of the study selection process.

A total of 314 articles were identified through a systematic search strategy conducted in electronic medical databases. After removing duplicate records (*n* = 93), the remaining 221 studies were subjected to preliminary screening by the researchers based on their titles and/or abstracts. As a result of this review, nine studies proceeded to the full‐text evaluation stage. Among the studies for which full‐text review was completed, nine articles were included in the quality assessment and met the inclusion criteria for the systematic review. However, of these nine studies, only five provided sufficient quantitative data to be included in the meta‐analysis. The remaining four studies were therefore incorporated into the systematic review solely at the qualitative synthesis stage (Figure 1).

### 3.2. Quality Assessment

The JBI checklist for cross‐sectional studies was used to assess the methodological quality of the cross‐sectional studies. According to the established criteria, four studies (44.44%) were classified as high quality [10, 17, 19, 32], while five studies were found to have moderate methodological quality [20–22, 33, 34] (Figure 1). Following the quality assessment, nine studies were included in the systematic review.

### 3.3. Characteristics of Included Studies

The included studies were published between 2021 and 2024 (*n* = 9). A total of 1877 healthcare professionals were evaluated across these studies. Among them, 402 were family physicians, 1050 were nurses, 202 were nurse academicians, and 223 were healthcare professionals working in various other fields. The majority of the studies (*n* = 1648) were conducted in Türkiye. Some of the studies focused specifically on healthcare professionals’ levels of AI anxiety [20, 21, 32], while others examined associated factors related to AI anxiety (*n* = 6) (Table 3).

**TABLE 3:** Characteristics of included studies.

Author (Year), country	Study design	Participants	Sample size	Data collection tools	Results
Başer [20], Turkey	A descriptive and cross‐sectional.	Family Physicians.	402	• Artificial concern scale	The mean total score of AIAS was 76.30 ± 27.87, the learning subdimension mean score was 24.83 ± 11.46, the job change subdimension mean score was 21.51 ± 8.68, and the sociotechnical subscale mean score was 18.95 ± 6.44. The mean score of the artificial intelligence configuration subdimension was 6.44, and 10.99 ± 5.96.
				• AIAS 21 item	In the comparative analysis of the scale, it was seen that there was no meaningful difference between genders, experience and regions. Despite there was a difference in artificial intelligence configuration based on gender, since the effect was too low, it was dismissed as unimportant.
Çobanoğlu [21], Turkey	A descriptive and cross‐sectional.	Nurses.	120	• Nurse Information From (NIF)	The mean score of the nurses’ AIAS was 43.36 ± 11.13, the score of the learning subdimension was 11.75 ± 3.73, the job replacement subdimension was 10.55 ± 3.56, the sociotechnical blindness subdimension was 12.60 ± 3.40, the AI configuration subdimension was 8.04 ± 3.00. It was determined that there was a difference between the educational status of the nurses, their knowledge of AI technologies, the effect of AI technologies in patient care and their AI anxiety levels (*p* < 0.05).
				• AIAS, 16 item	
Ergin [32], Turkey	Single group pre‐ and post‐test quasi‐experimental.	Nurses. Nurses working in the operating room.	47	Artificial intelligence knowledge questionnaire	The mean score of the nurses’ AI anxiety scale was 46,14 ± 11,79, the score of the learning subdimension was 12,28 ± 4,73, the job replacement subdimension was 12,28 ± 3,76, the sociotechnical blindness subdimension was 13,25 ± 3,04, the AI configuration subdimension was 8,31 ± 2,86.
				AIAS, 16 item	
Maraş [17], Turkey	A descriptive and cross‐sectional.	158 intern students and 167 surgical nurses.	167	• Questions form regarding humanoid nurse robots and artificial intelligence health technologies	The total scores on the Artificial Intelligence Anxiety Scale for nurses and nursing students are 73.089 ± 31.667 and 73.624 ± 28.029, respectively. The total scores on the Artificial Intelligence Readiness Scale for nurses and nursing students are 71.736 ± 15.064 and 72.183 ± 13.714, respectively. No statistically significant difference was found between the mean scores of AIAS among nurses concerning gender, education level and years of work experience (*p* > 0.05).
				• AIAS 21 item	
				• Medical Artificial Intelligence Preparedness Scale (MAIPS)	
Subaşı [10], Turkey	A descriptive, correlational and cross‐sectional	Nurses actively working in pediatric clinics.	170	• Artificial intelligence literacy scale	The study indicated significant positive correlations between paediatric nurses’ age and their AIAS scores (*r* = 0.226; *p* < 0.01) and significant negative correlations between paediatric nurses′ age and their AILS (*r* = −0.192; *p* < 0.05) and GAAIS scores (*r* = −0.152; *p* < 0.05). There is a statistically significant positive weak correlation between age and the Learning and Job Replacement subscales of AIAS (*p* < 0.05). Conversely, there is a statistically significant negative weak correlation between age and the Evaluation subscale of AILS, and positive GAAIS (*p* < 0.01).
				• AIAS, 21 item	
				• The general attitudes towards artificial intelligence scale	
Tarsuslu [33], Turkey	A descriptive, correlational and cross‐sectional	Nurses.	439	• Digital leadership scale	It was determined that 29.8% of the nurses had a good relationship with technology, 66.3% knew about using artificial intelligence in health, and 27.3% wanted it to be more involved in their lives. It was determined that nurses’ perceptions of digital leadership were at a medium level of 46.9% and a high level of 41.7%, 82.7% had a positive attitude towards artificial intelligence, and 82.7% had low or medium level anxiety when their artificial intelligence anxiety status was examined. There was a significant and negative relationship between digital leadership and AI anxiety (*r* = −0.434; *p* < 0.01), a significant and positive relationship between digital leadership and AI attitude (*r* = 0.468; *p* < 0.01), and a significant and negative relationship between AI attitude and AI anxiety (*r* = −0.629; *p* < 0.01). Finally, it was determined that nurses’ perception of digital leadership indirectly affected AI anxiety through AI attitude (*β* = −0.230, 95% CI [‐0.298, −0.165]).
				• Artificial intelligence attitude scale	
				• AIAS, 16 item	
Ünal [19] Turkey	A descriptive and cross‐sectional.	Neonatal nurses.	107	• AIAS, 16 item	There was a statistically significant moderate negative correlation between participants’ AIAS scores and MAIRS scores (*r* = −0.549). AIAS scores differed statistically significantly by age, education level, experience in neonatal care, knowledge about artificial intelligence, favouring the existence of AI‐based technologies in neonatal clinics, and anxiety about artificial intelligence (*p* < 0.05). MAIRS scores differed statistically significantly (*p* < 0.05) by education level, having knowledge about artificial intelligence, favouring the existence of AI‐based technologies in neonatal clinics, and anxiety about artificial intelligence.
				• Medical Artificial Intelligence Readiness Scale (MAIRS)	
Weber [22], Germany	A mixed‐methods study.	Medical professionals: physician, nurse, paramedic, and/or in the emergency medical services.	223	• AIAS, 21 item	The results suggest that this fear is driven by little or moderate knowledge about AI.
				• Specific consumer knowledge scale	
Yiğit [34], Turkey	A descriptive and cross‐sectional.	Nurse academician.	202	• AIAS 16 item	It was determined that the mean score of the academicians on the AIAS was 57.59 ± 8.84. All participants stated that they had heard of the concept of artificial intelligence before. It was determined that there was a significant relationship between the academicians’ receiving training on artificial intelligence, their belief that artificial intelligence will affect the nursing profession in the future, and their mean score on the AIAS.

The studies utilized both the original 21‐item version and both the structured 16‐item version of the AIAS. In the study conducted by Başer et al. [20] using the original 21‐item form of the AIAS with family physicians, the mean total score was reported as 76.30 ± 27.87, and no significant differences were found between the scale scores and variables such as experience, gender, and geographical region [20]. Similarly, in the study conducted by Maraş et al. [17], the mean AIAS score of 167 surgical nurses was reported as 73.08 ± 31.66, and no significant relationship was found between the scale scores and variables such as gender, professional experience, or education level [17]. On the other hand, in a study conducted by Subasi et al. [10] with nurses working in a pediatric clinic, although the mean total score was not reported, a positive correlation was found between AIAS scores and age [10]. In a mixed‐method study involving physicians, nurses, and various other healthcare professionals, although AIAS scores were not reported, it was noted that a lack of knowledge about AI was associated with higher levels of AI‐related anxiety [22]. In studies using the structured 16‐item version of the AIAS, Çobanoğlu et al. [21] reported that nurses had a mean AIAS score of 43.36 ± 11.13, while Ergin et al. [32] found that operating room nurses had a mean score of 46.14 ± 11.79. Tarsuslu et al. [33], without reporting a total score, identified a significant negative correlation between attitudes toward AI and AI anxiety (*r* = −0.629; *p* < 0.01). In a study by Ünal et al. [19] conducted with neonatal nurses, AIAS scores were found to differ significantly based on variables such as age, education level, experience in neonatal care, having knowledge about AI, support for AI‐based technologies in neonatal units, and anxiety about AI. In a study conducted with nurse academicians, the mean AIAS score was reported as 57.59 ± 8.84, and a significant relationship was found between these scores and their belief that AI would impact the nursing profession in the future.

### 3.4. AIAS Findings

**FIGURE 2:**
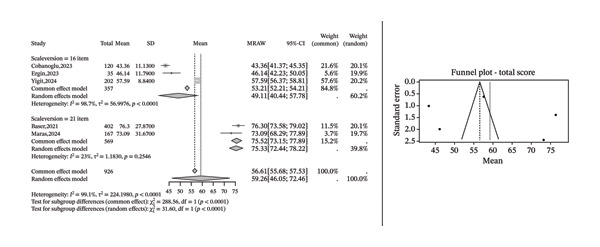
Forest plot and funnel plot of the total AI anxiety scale scores.

A meta‐analysis was conducted based on the mean total and subdimension scores of the AIAS reported in the included studies. Four of the reviewed studies were excluded from the meta‐analysis because they did not report total or subdimension scores of the AIAS [10, 19, 22, 33]. The meta‐analysis included five studies that reported measurement scores [17, 20, 21, 32, 34], with a total combined sample size of 926 healthcare professionals. Since both the 16‐item and 21‐item versions of the scale were used across studies, a subgroup analysis was conducted. In the first subgroup, comprising studies using the 16‐item version, data from 357 participants were analyzed; in the second subgroup, using the 21‐item version, data from 569 participants were included. The meta‐analysis revealed that the mean AIAS score was 59.26 (95% CI: 40.54–77.97), indicating a moderate level of AI‐related anxiety among healthcare professionals. The meta‐analysis also revealed a statistically significant and high level of heterogeneity among the studies (*I* ^2^ = 99.1%, *τ* ^2^ = 224.20, *p* < 0.0001) (Figure 2).

**FIGURE 3:**
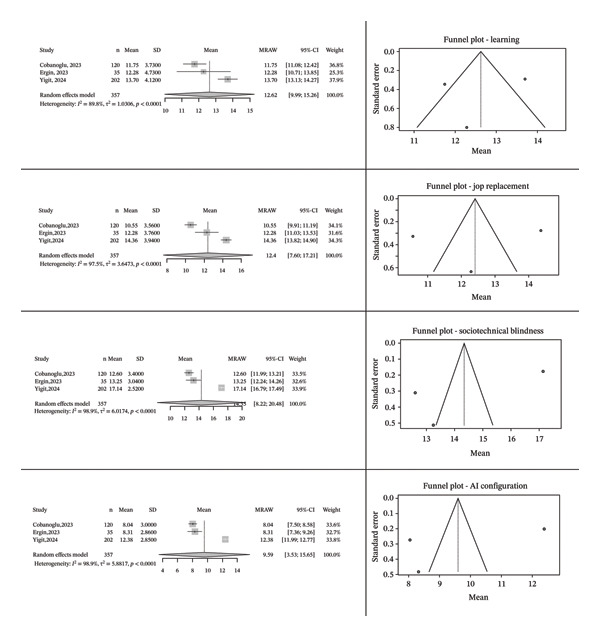
Funnel plot of AIAS subdimensions.

In the meta‐analysis, only one study addressed the subdimensions of the 21‐item version of the AIAS, while three other studies [10, 17, 22] did not report subdimension scores. Additionally, three studies using the 16‐item version [19, 33] also did not provide subdimension data, and were therefore excluded. The meta‐analysis was completed with the inclusion of three studies that reported subdimension scores [21, 32, 34]. In the meta‐analysis of the ‘Learning’ subdimension, data from a total of 357 participants were evaluated. Using a random‐effects model, the mean score for this subdimension was calculated as 12.62 (95% CI: 9.99–15.26). The analysis revealed a high level of heterogeneity (*I* ^2^ = 89.8%, *τ* ^2^ = 1.0306, *p* < 0.0001). These results indicate that healthcare professionals experience a moderate level of anxiety regarding the impact of AI on learning, although there is substantial variation between studies. In the meta‐analysis of the ‘Job Replacement’ subdimension, the mean score was found to be 12.40 (95% CI: 7.60–17.21). The level of heterogeneity was determined to be *I* ^2^ = 97.5%, *τ* ^2^ = 3.6473, and *p* < 0.0001. This finding indicates that healthcare professionals also experience a moderate level of anxiety regarding the impact of AI on the workforce. For the ‘Sociotechnical Blindness’ subdimension, the mean score was calculated as 14.35 (95% CI: 8.22–20.48). Heterogeneity was found to be *I* ^2^ = 98.9%, *τ* ^2^ = 6.0174, and *p* < 0.0001. These results suggest that healthcare professionals experience a moderate level of anxiety related to sociotechnical blindness regarding AI. Finally, for the ‘AI Configuration’ subdimension, the mean score was found to be 9.59 (95% CI: 3.53–15.65), with a heterogeneity level of *I* ^2^ = 98.9%, *τ* ^2^ = 5.8817, and *p* < 0.0001. These findings indicate that healthcare professionals have moderate levels of anxiety concerning the technical configuration of AI. When differences between subdimensions were examined, the test results revealed a statistically significant difference (*χ* ^2^ = 73.81, df = 4, *p* < 0.0001) (Figure 3).

### 3.5. Publication Bias

In the meta‐analysis results, the Egger’s test was conducted to assess the presence of publication bias. The test yielded a t‐value of 0.34 and a *p*‐value of 0.7537, indicating no significant evidence of publication bias. Additionally, when the bias estimate in Egger’s test (bias estimate = 4.0489) was evaluated together with the standard error (SE = 11.7790), the result indicated that this estimate was not statistically significant, suggesting no substantial publication bias. Similarly, the Begg’s test yielded a high *p*‐value (0.6242), also indicating no evidence of publication bias. The z‐value obtained from Begg’s test was 0.49, further suggesting that the observed effects across studies were not primarily influenced by publication bias. The bias estimate in Begg’s test was 2.0000, and when considered with its standard error (SE = 4.0825), these findings also confirmed that the risk of bias was negligible.

### 3.6. Sensitivity Analysis

The ‘leave‐one‐out’ approach was employed for the sensitivity analysis. The exclusion of the studies by Başer [20], Çobanoğlu [21], Ergin [32], Maraş [17], and Yiğit [34] did not result in any substantial changes in the overall mean values. However, the exclusion of each individual study did have an impact on heterogeneity. Notably, when the study by Yiğit [34] was removed, heterogeneity metrics increased (*I* ^2^ = 99.3%; *τ* ^2^ increased), indicating that this study had a reducing effect on overall heterogeneity. In other words, the inclusion of Yiğit’s [34] study contributed to a lower level of heterogeneity in the meta‐analysis. Although fluctuations in heterogeneity were observed depending on the excluded study, the overall findings of the sensitivity analysis showed that none of the included studies had a significant influence on the meta‐analysis results. This suggests that the overall findings of the meta‐analysis are robust, reliable, and not overly dependent on any single study. These results from the sensitivity analysis support the generalizability and methodological rigor of the research.

## 4. Discussion

This meta‐analysis focused on studies examining healthcare professionals’ levels of anxiety related to AI, providing insights into the overall AI anxiety levels and their subdimensions. To the best of our knowledge, this is the first meta‐analysis to systematically address AI‐related anxiety among healthcare professionals.

A total of five studies were included in this meta‐analysis. The primary reason for the exclusion of other studies was that they focused on correlation and regression analyses without reporting total or subdimension scores of the scale. Additionally, one study was excluded from the subdimension analysis due to the use of a different number of items in the AIAS. The differences between the scale versions used in the studies emerged as an important factor to consider when interpreting the findings. The need for subgroup analyses due to the use of different versions highlights the necessity of standardizing such measurement instruments.

These results of the meta‐analysis indicate that healthcare professionals experience a moderate level of anxiety related to AI. This may suggest that, when confronted with this new system, healthcare professionals experience internal tensions such as uncertainty, feelings of inadequacy, or loss of control. Moderate levels of anxiety may also be associated with being unprepared, lack of knowledge, the need for training, or anxiety regarding the ethical and social implications of AI integration. Although anxiety may trigger negative emotions, when experienced at mild to moderate levels, it can also serve as a motivating factor that encourages action and supports engagement [23]. This suggests that anxiety may, in fact, be beneficial for healthcare professionals. It is anticipated that moderate levels of anxiety may lead individuals to become more attentive to environmental changes, actively seek information, take initiative, and make more informed decisions. However, if this level of anxiety is not appropriately addressed, it may lead to resistance against technological innovations or hinder the integration process. Therefore, to facilitate healthier adaptation of healthcare professionals to AI‐related developments, it is crucial to provide adequate institutional support, offer comprehensive training programs, and encourage their active participation in implementation processes. However, the interpretation of this finding is limited due to the high heterogeneity among the studies.

In the study conducted by Weber et al. [22], it was reported that AI‐related anxiety stemmed from low to moderate levels of knowledge. Similarly, other studies have emphasized the association between limited knowledge of AI technologies and increased AI anxiety [19, 21, 33]. The study by Başer et al. with family physicians further supports these findings, as it reported that participants’ low anxiety levels were associated with their technological knowledge [20]. However, in a quasi‐experimental study conducted with operating room nurses who received training on the use of AI and robotic nursing technologies, anxiety levels were found to have increased following the intervention. This finding suggests that an increase in knowledge does not always lead to a reduction in anxiety; rather, heightened awareness of personal deficiencies may, in some cases, contribute to increased anxiety [32]. It can be stated that the learning process should be addressed not only through knowledge transfer but also by considering its cognitive and emotional dimensions. In the study conducted by Maras et al., a significant relationship was found between AI anxiety and age, years of experience, and gender; anxiety decreased with younger age and fewer years of experience, while female participants exhibited higher levels of anxiety [17]. These findings indicate that sociodemographic variables also influence anxiety levels, emphasizing that age, professional experience, and gender are key determinants in this process. When planning AI‐related training and support programs, it is crucial to adopt individualized approaches that consider personal characteristics such as age, years of experience, and gender, given their significant role in technological adaptation.

This study has several limitations. First, the number of studies included in the meta‐analysis was limited. Second, the high heterogeneity across studies, particularly due to differences in measurement tools and study populations, may limit the generalizability of the findings. Third, the exclusion of studies that did not report sufficient quantitative data may have resulted in the omission of relevant evidence.

## 5. Limitations

This study has several limitations that should be considered. The inability to include all relevant studies in the meta‐analysis and the high heterogeneity observed across the analyses limit the generalizability of the findings. In addition, variations in sample characteristics and study designs across the included studies may have influenced the results. Although methodological consistency was enhanced by prioritizing the use of the AIAS, differences in scale versions may have affected comparability.

## 6. Recommendations

Based on the findings of this study, several recommendations can be made. Standardization of measurement tools is essential to accurately assess healthcare professionals’ anxiety levels toward AI and to improve comparability across studies. Future research should focus on more homogeneous samples and adopt robust methodological designs. Additionally, to reduce healthcare professionals’ anxiety related to AI technologies, the implementation of professional training programs, institutional awareness‐raising initiatives, and the development of supportive policies are of great importance. Furthermore, longitudinal and intervention‐based studies are recommended to better understand and manage AI‐related anxiety.

## 7. Conclusion

This meta‐analysis examined healthcare professionals’ anxiety levels toward AI and found that they experience a moderate level of anxiety toward AI technologies. These findings highlight the growing importance of addressing AI‐related concerns among healthcare professionals as the integration of AI into healthcare systems continues to expand.

## Author Contributions

All authors contributed to the conception and design of the study. Hilal Kahraman, Hatice Yüceler Kaçmaz, and Seda Akutay performed conception of the work, data collection, and analysis. Hilal Kahraman wrote the initial draft of the manuscript, and all authors provided comments on subsequent versions.

## Funding

This research did not receive any specific grant from funding agencies in the public, commercial, or non‐profit sectors.

## Disclosure

All authors read and approved the final manuscript.

## Conflicts of Interest

The authors declare no conflicts of interest.

## Data Availability

The data that support the findings of this study are available from the corresponding author upon reasonable request.
